# A three-source capture-recapture estimate of the number of new HIV diagnoses in children in France from 2003–2006 with multiple imputation of a variable of heterogeneous catchability

**DOI:** 10.1186/1471-2334-12-251

**Published:** 2012-10-10

**Authors:** Vanina Héraud-Bousquet, Florence Lot, Maxime Esvan, Françoise Cazein, Corinne Laurent, Josiane Warszawski, Anne Gallay

**Affiliations:** 1 Institut de Veille Sanitaire, Département des maladies infectieuses, St Maurice, France; 2 Inserm CESP U1018, Le Kremlin-Bicêtre. AP-HP, Service de santé publique, Le Kremlin-Bicêtre, France; 3 Inserm CESP U1018, Le Kremlin-Bicêtre. Université Paris-Sud, Le Kremlin Bicêtre. AP-HP, Service de santé publique, Le Kremlin-Bicêtre, France

## Abstract

**Background:**

Nearly all HIV infections in children worldwide are acquired through mother-to-child transmission (MTCT) during pregnancy, labour, delivery or breastfeeding. The objective of our study was to estimate the number and rate of new HIV diagnoses in children less than 13 years of age in mainland France from 2003–2006.

**Methods:**

We performed a capture-recapture analysis based on three sources of information: the mandatory HIV case reporting (DOVIH), the French Perinatal Cohort (ANRS-EPF) and a laboratory-based surveillance of HIV (LaboVIH). The missing values of a variable of heterogeneous catchability were estimated through multiple imputation. Log-linear modelling provided estimates of the number of new HIV infections in children, taking into account dependencies between sources and variables of heterogeneous catchability.

**Results:**

The three sources observed 216 new HIV diagnoses after record-linkage. The number of new HIV diagnoses in children was estimated at 387 (95%CI [271–503]) from 2003–2006, among whom 60% were born abroad. The estimated rate of new HIV diagnoses in children in mainland France was 9.1 per million in 2006 and was 38 times higher in children born abroad than in those born in France. The estimated completeness of the three sources combined was 55.8% (95% CI [42.9 – 79.7]) and varied according to the source; the completeness of DOVIH (28.4%) and ANRS-EPF (26.1%) were lower than that of LaboVIH (33.3%).

**Conclusion:**

Our study provided, for the first time, an estimated annual rate of new HIV diagnoses in children under 13 years old in mainland France. A more systematic HIV screening of pregnant women that is repeated during pregnancy among women likely to engage in risky behaviour is needed to optimise the prevention of MTCT. HIV screening for children who migrate from countries with high HIV prevalence to France could be recommended to facilitate early diagnosis and treatment.

## Background

Nearly all HIV infections that occur worldwide in children are acquired through mother-to-child transmission (MTCT) during pregnancy, labour, delivery or breastfeeding. Estimates have shown that there were nearly 430,000 new paediatric infections worldwide in 2008 [1]. Nearly all such infections can be prevented through MTCT prevention programmes.

In France, the risk of HIV transmission from mother to child has been dramatically reduced since the end of the 1980s through the prophylactic use of antiretroviral therapy (ART) during pregnancy and the administration of ART drugs to the baby during the first weeks of life. Currently, the risk of HIV transmission from mother to child is approximately 1% [2]. The early diagnosis of HIV infection during pregnancy and early treatment of the mother allow for the effective prevention of MTCT. In France, the national policy since 1993 has been to offer universal voluntary HIV testing in the first trimester of pregnancy. Moreover, it was recommended in 2002 to repeat HIV testing during pregnancy in women at high risk of HIV transmission [3].

Of the 150,000 people living in France with HIV, it is estimated that approximately 1,500 are children. In newborns, ten to fifteen new HIV infections are diagnosed each year [4], an estimate based on the application of the MTCT rate of 1% to the number of HIV-positive pregnant women delivering each year in France. However, this estimate does not take into account infections in children born abroad (in high endemic countries) who are diagnosed after their arrival in France. Foreign-born populations account for 5.3 million individuals, which equates to approximately 8.3% of the total population in France. Among these migrants, 5.8% are children under 13 years of age, and 40% are living in the Paris area [5]. Migrants are typically born in Europe (38.4%), North Africa (30.1%), Asia (14.0%), or sub-Saharan Africa (12.3%) [6]. Currently, there are no diagnostic testing guidelines for children except for those born from HIV seropositive mothers. Targeted screening is recommended for migrants who originate from countries with a high prevalence of HIV; however, this recommendation does not strictly include children [7].

In this paper, we estimated the total number and rate of new HIV diagnoses in children less than 13 years of age in mainland France between 2003–2006 using capture-recapture methods. We used three data sources: the mandatory HIV case reporting (DOVIH), the ANRS French Perinatal Cohort (Enquête Périnatale Française) (EPF) and the HIV laboratory surveillance system (LaboVIH). We also assessed the completeness of the 3 sources along with the combined completeness (case-ascertainment).

## Methods

The capture-recapture method estimates the total number of cases of a disease after matching cases reported in at least two sources [8].

### Case definition

Cases were defined as all new HIV infections in children under 13 years of age, according to microbiological criteria [4], that were diagnosed in mainland France (the part of France located in Europe) during the 2003–2006 period.

### Description of the three data sources

#### The mandatory HIV case reporting (DOVIH)

The mandatory HIV case reporting system was implemented in 2003 by the French Institute for Public Health Surveillance (InVS) to follow the epidemic trends of HIV and to describe the characteristics of HIV infections in newly diagnosed individuals [9]. For adults, HIV mandatory notifications are initiated by microbiologists and then completed by clinicians. For children under 13 years of age, case reporting is performed only by paediatricians. All HIV-positive cases are notified using an assigned unique anonymous code that allows for the detection of duplicates. To take into account reporting delays, all notifications through March 31^st^, 2010 were selected for the study.

#### The ANRS French Perinatal Cohort (ANRS-EPF CO1/CO10/CO11)

Since 1984, the French Perinatal Cohort, supported by the French National Agency for AIDS Research (ANRS), has prospectively collected data on HIV-infected pregnant women and their children in approximately one hundred centres throughout France [2]. The coverage of the cohort was estimated at 70% of cases throughout France. The objectives of this cohort study are to identify factors associated with HIV MTCT, to evaluate tolerance to ART prophylaxis, and to assess the prognosis of paediatric HIV infection. Informed consent was obtained from all of the mothers. Since 2005, the inclusion criteria were extended to all children <13 years of age diagnosed with HIV and born to mothers who were not included in the EPF, with parental consent. For these children, data were collected retrospectively for 2003 and 2004 and prospectively since 2005. Duplicates were deleted. The cases were selected based on a database that was updated in April 2008.

#### The HIV laboratory surveillance (LaboVIH)

Since 2001, the InVS has implemented a national surveillance of the HIV testing activity in France. The number of HIV tests performed and the number of new HIV-positive confirmed diagnoses are collected from 4,200 French microbiological laboratories each year [10]. The participation rate of this laboratory surveillance is approximately 85%.

Laboratories that reported at least one new HIV diagnosis in children less than 13 years of age from 2003 to 2006 were asked to complete a questionnaire to collect individual information for each paediatric diagnosis. Duplicate notifications were deleted.

### Identification of common cases among sources

Because no common identification code was available among the three sources, algorithms were established using variables common to all three sources to identify common cases. Year of birth, sex, reference hospital (or district number) and date of diagnosis (or date of the first medical care) were available in all three sources. The algorithm that detected common cases between the DOVIH and EPF sources also included the maternity of birth for children born in France, or the country of birth for children born abroad, the mother’s country of origin and the vital status of the children. The identification of common cases among the sources was performed with the SQL procedure in SAS© version 9.1 and was completed by a manual verification of matched records.

### Imputation of the variable “country of birth” in the source LaboVIH

We wanted to estimate the total number of new HIV diagnoses according to the place of birth: “born in France” or “born abroad”. This binary variable was not collected in the LaboVIH source. However, this variable was collected in the DOVIH and EPF sources. Therefore, we were able to obtain the place of birth for the cases in LaboVIH that matched the two other sources of information (DOVIH and the EPF). The variable was missing in two cases in DOVIH and was unavailable for 66/126 cases globally (30.6%). We estimated the missing values through a multiple imputation (MI) method, in which the distribution of the observed data is used to estimate a set of plausible values for the missing observations [11]. Multiple data sets were created, and an estimate was calculated for each imputed data set. The estimates were then combined to calculate overall estimates, variances and confidence intervals.

The applied MI method was multiple imputation by chained equations using STATA's user-written program *ice* (STATA ® 11.0, Stata Corporation, College Station, Texas, USA) [12,13]. The variables “age” (continuous), “region of diagnosis” (categorical) and “year of diagnosis” (categorical) contained no missing values and were used as predictors in the imputation model. One hundred imputed databases were generated.

### Capture-recapture estimates

The reliability of the estimates depended on the following underlying assumptions: (1) identification of all and only true common cases, (2) closed population, (3) independence between sources and (4) capture homogeneity [8]. Two sources are independent if the probability of a case being reported in one source does not depend on its probability of being reported in the other source. For analyses involving three or more sources, the independence assumption is not crucial because interaction terms can be incorporated into regression models to adjust for source dependencies; however, in these cases, highest-order independence has to be assumed. Homogeneity of capture is fulfilled when the probability of a case being reported in a source is the same for all cases or, more simply, when the probability of registration does not depend on the characteristics of the case (i.e., age, sex, place of birth etc.). This probability may vary from one source to another or be constant overall [8].

Dependence between sources was first assessed by comparing the estimates provided by each pair of sources [14,15] and calculating the odds ratio (95% CI) between the two sources, as proposed by Wittes [8].

A preliminary three-source analysis was performed by fitting eight log-linear models to the data arranged in a 2^3^ contingency table, according to the presence or absence of each case in each source. The dependent variable for each model was the logarithm of the number of cases in each of the 7 non-empty cells of the contingency table. These preliminary analyses assumed homogeneity of capture within each source and were performed using STATA’s user-written program “recap” [16], a STATA module providing standard three-source capture-recapture analyses without covariates. The confidence interval estimates for the population size were computed according to a goodness-of-fit based method proposed by Regal and Hook [17].

Three variables of potential heterogeneous catchability were considered: place of birth (born in France; born abroad), region of diagnosis (Paris area; other regions), and year of diagnosis (2003 to 2006). The data were then arranged in a 2^3^x2x2x4 contingency table. Log-linear models were fitted via the STATA ‘glm’ command, which specified a logarithmic link and a Poisson distribution. Stratified analyses were performed according to the three variables of heterogeneous catchability. The log-linear models included two-way interactions between sources, between sources and each variable of catchability, and between the variables of catchability, when applicable. Log-linear modelling was jointly performed for the 100 imputed data sets using the STATA 11.0 analysis module “mi estimate” applying Rubin’s rules.

Population size estimates, calculated as a sum of exponentiated regression coefficients, were obtained through commands specific to MI. Their respective variances were estimated using the delta method. The confidence intervals (CI) were computed using Student’s t-statistics with degrees of freedom specific to each coefficient, depending both on the number of imputations and on the proportion of missing values.

Classically, in capture-recapture studies, the choice of the final model is based on the likelihood ratio test statistic (*G*^*2*^), the Akaike Information Criterion (AIC) and the Bayesian Information Criterion adapted by Draper (DIC), which are functions of the likelihood ratio statistic [18,19]. AIC and DIC criteria were derived for each imputed data set according to the following formulas: *AIC* = *G*^2^ − 2(*df*) and *DIC* = *G*^2^ − (ln(*N*_*obs*_/2*π*)) · (*df*), where df is the number of degrees of freedom associated with any model.

The naïve approach that averages the likelihood ratio statistic over the imputed data sets does not provide accurate p-values [20]. The pooled likelihood ratio test statistic and its corresponding p-value were calculated using the Meng and Rubin approach [21], recently illustrated by Marshall *et al.*[22]. Each log-linear model was constrained to the regression coefficients obtained from the joint analysis (i.e., the average over the 100 imputed data sets, according to Rubin’s rules). The AIC and DIC estimates were the average of the 100 AICs and DICs. We selected the most parsimonious model among the models with a goodness-of-fit p-value >0.05, and with the lowest AIC and DIC values. We also considered the relevance of including variables of heterogeneous catchability in the model, both from an epidemiological and a public health point of view.

The completeness for each source was estimated by dividing the number of new HIV diagnoses reported in each source by the total number estimated by the final log-linear model. The completeness was also calculated for each stratum of “place of birth”, “year of diagnosis” and “region of diagnosis”.

The annual rate of new HIV diagnoses was the estimated number of new HIV diagnoses divided by the size of the population of children under 13 years old living in mainland France up to December 2007 [23]. The rate was also calculated according to the place of birth, using the number of children less than 13 years of age born in France or abroad.

Access to the 3 databases was authorised by the French Commission Nationale de l' Informatique et des Libertés (CNIL). No ethical approval was required for this research.

## Results

### Cross-matches

**Figure 1:**
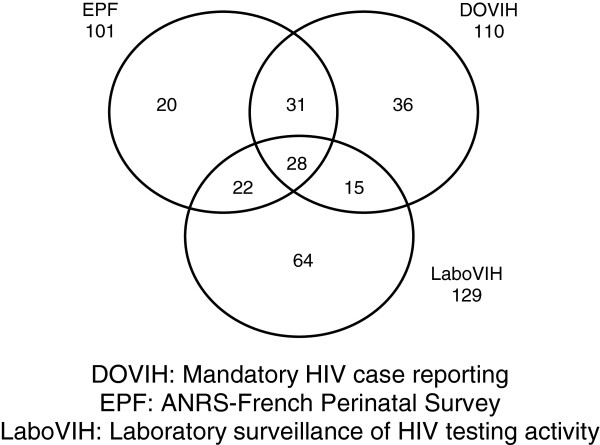
Distribution of new HIV diagnoses in the children identified by the three sources (N=216).

The three sources reported 216 new HIV diagnoses in children under 13 years old in mainland France between January 1st, 2003 and December 31st, 2006 (Figure 1).

### Capture-recapture estimates

When performing two-source capture-recapture analysis, the estimate of the number of new HIV diagnoses provided by matching the sources DOVIH and EPF (N_est_ = 188; 95%CI [171 – 206]) was lower than the estimate provided by matching LaboVIH and EPF (N_est_ = 261; 95%CI [224–297]) or LaboVIH and DOVIH (N_est_ = 330; 95%CI [272–389]), suggesting a positive dependence between the sources DOVIH and EPF. The Wittes odds ratio confirmed the dependence between the sources DOVIH and EPF (OR = 5.4; 95%CI [2.5-12.1]) and suggested a positive dependence between LaboVIH and EPF (OR = 2.2; 95%CI [1.0-4.8]).

Preliminary log-linear modelling using the three sources and including the dependencies between sources provided an estimate of 369 (95%CI [294–521]) new HIV diagnoses during the 2003–2006 period (Table 1). This model took into account two dependencies between sources (DOVIH*EPF and EPF*LaboVIH).

**Table 1:** Preliminary log-linear analyses assuming homogeneity of capture within each source

**Log-linear models**	$\hat{n}$	$\hat{N}$	**95% CI**	**df**	**G**^**2**^	**p**	**AIC**	**DIC**
Dependencies between sources								
LaboVIH*DOVIH, LaboVIH*EPF, DOVIH*EPF	126	342	259,573	0	0	1	0	0
LaboVIH*DOVIH, LaboVIH*EPF	23	239	225,263	1	18.83	<10^-4^	16.83	16.89
LaboVIH*DOVIH, DOVIH*EPF	58	274	243,331	1	3.78	0.05	1.78	1.84
LaboVIH*EPF, DOVIH*EPF	153	369	294,521	1	0.24	0.63	−1.76	−1.71
LaboVIH*DOVIH, EPF	29	249	234,272	2	18.49	<10^-4^	14.49	14.6
LaboVIH*EPF, DOVIH	51	267	245,300	2	30.12	<10^-4^	26.12	26.23
DOVIH*EPF, LaboVIH	85	301	268,349	2	5.96	0.05	1.96	2.07
LaboVIH, DOVIH, EPF	49	265	246,292	3	30.2	<10^-4^	24.20	24.36

DOVIH: Mandatory HIV case reporting; EPF: ANRS French Perinatal Cohort; LaboVIH: Laboratory surveillance of HIV testing activity.

$\hat{n}$: Estimate of the number of diagnoses not reported to any source; $\hat{N}$: Estimate of the number of diagnoses; 95% CI: 95% confidence interval for $\hat{N}$_;_ df: number of degrees of freedom; G^2^: deviance statistic; p: p-value of the deviance goodness-of-fit test; AIC: Akaike Information Criterion; DIC: Draper Information Criterion.

When considering the dependencies with variables of catchability, the model with the lowest AIC and a likelihood ratio test with p>0.05 provided an estimate of 387 (95%CI [271–503]) new HIV diagnoses during the same period (Table 2). This model (model 7) included two interactions between sources, and interactions between sources and variables of catchability (DOVIH*place of birth, EPF*place of birth, DOVIH*region of diagnosis, EPF*region of diagnosis, LaboVIH* region of diagnosis, and EPF*year of diagnosis). The estimated annual number of new HIV diagnoses decreased over time from 108 in 2003 to 89 in 2006 (Table 3).

**Table 2:** Log-linear analyses incorporating variables of potential heterogeneous catchability

**Log-linear models**	$\hat{n}$	$\hat{N}$	**95% CI (**$\hat{N}$**)**		**df**	**G**^**2**^	**p**	**AIC**	**DIC**
Model 1: DO*EPF, LABO*EPF, EPF*place	154	370	270	469	100	145,75	0,00	−54,25	−142,31
Model 2: DO*EPF, LABO*EPF, EPF*region	154	370	270	469	100	133,74	0,01	−66,26	−154,32
Model 3: DO*EPF, LABO*EPF, EPF*année, Labo*année	154	370	270	469	95	141,78	0,00	−48,22	−131,88
Model 4: DO*EPF, LABO*EPF, EPF*place, EPF*region, DO*region	147	363	266	460	97	125,12	0,03	−68,88	−154,30
Model 5: DO*EPF, LABO*EPF, EPF*place, EPF*year	131	347	260	434	94	132,81	0,00	−55,19	−137,96
Model 6: DO*EPF, LABO*EPF, EPF*region, DO*region, EPF*year	141	357	261	452	96	121,31	0,02	−64,69	−146,59
Model 7: DO*EPF, LABO*EPF, DO*place, EPF*place, DO*region, Labo*region, EPF*region, EPF*year	171	387	271	503	93	112,72	0,07	−73,28	−155,17
Model 8: DO*EPF, LABO*EPF, DO*place, EPF*place, DO*region, Labo*region, EPF*region	171	387	271	503	96	112,48	0,05	−73,52	−158,06

DO: Mandatory HIV case reporting; EPF: ANRS French Perinatal Cohort; Labo: Laboratory surveillance of HIV testing activity.

$\hat{n}$: Estimate of the number of diagnoses not reported to any source; $\hat{N}$: Estimate of the number of diagnoses; 95% CI: 95% confidence interval for $\hat{N}$_;_ df: number of degrees of freedom; G^2^: deviance statistic; p: p-value of the deviance goodness-of-fit test; AIC: Akaike Information Criterion; DIC: Draper Information Criterion

Place: place of birth; region: region of diagnosis; year: year of diagnosis.

**Table 3:** Estimates of completeness of each source (model 7)

**Strata**			**Total**			**DOVIH**			**EPF**			**LaboVIH**		
	$\hat{N}$	**(95% CI)**	***N***_***obs***_	**Compl(%)**	**(95% CI)**	***N***_***obs***_	**Compl(%)**	**(95% CI)**	***N***_***obs***_	**Compl(%)**	**(95% CI)**	***N***_***obs***_	**Compl(%)**	**(95% CI)**
Year of diagnosis														
2003	107	(72.4;142.7)	60	55.8	(42.0:82.9)	30	27.9	(21.0;41.4)	28	26.0	(19.6:38.7)	30	27.9	(21.0;41.4)
2004	99	(68.9;129.4)	59	59.5	(45.6;85.7)	35	35.3	(27.0;50.8)	32	32.3	(24.7;46.5)	35	35.3	(27.0;50.8)
2005	91	(62.4;120.9)	53	57.8	(43.8;85.0)	27	29.5	(22.3;43.3)	27	29.5	(22.3;43.3)	34	37.1	(28.1;54.5)
2006	88	(55.4;121.8)	44	49.7	(36.1;79.4)	18	20.3	(14.8;32.5)	14	15.8	(11.5;25.3)	30	33.9	(24.6;54.2)
Place of birth														
France	152	(100.3;204.9)	86	56.4	(42.0;85.7)	37	24.2	(18.1;36.9)	47	30.8	(22.9;46.8)	55	36.0	(26.8;54.8)
Foreign country	234	(158.9;309.9)	130	55.5	(42.0;81.8)	73	31.1	(23.6;45.9)	54	23.0	(17.4;34.0)	74	31.6	(23.9;46.6)
Region of diagnosis														
Paris area	198	(154.7;241.4)	139	70.2	(57.6;89.9)	79	39.9	(32.7;51.1)	79	39.9	(32.7;51.1)	82	41.4	(34.0;53.0)
Other regions	188	(101.0;276.8)	77	40.8	(27.8;76.2)	31	16.4	(11.2;30.7)	22	11.6	(7.9;21.8)	47	24.9	(17.0;46.5)
Total	387	(271;503)	216	55.8	(42.9;79.7)	110	28.4	(21.9;40.1)	101	26.1	(20.1;37.3)	129	33.3	(25.6;47.6)

DOVIH: Mandatory HIV case reporting; EPF: ANRS French Perinatal Cohort; LaboVIH: Laboratory surveillance of HIV testing activity.

$\hat{N}$: Estimate of the number of diagnoses; *N*_*obs*_: Number of diagnoses observed; Compl: Completeness; 95% CI: 95% confidence interval for completeness.

The estimated completeness of the combined three sources was 55.8% (CI 95% [42.9 – 79.7]), but varied according to the source (Table 3). The completeness of DOVIH (28.4%) and EPF (26.1%) were lower than that of LaboVIH (33.3%). The completeness had slightly decreased since 2004 in both DOVIH and EPF, particularly during the last year (2006). The completeness was greater in the Paris area than in other regions in the three sources and was greater for children born in France, compared with abroad, in the sources EPF and LaboVIH.

Based on the estimated number of new diagnoses obtained in Table 3, the rate of new HIV diagnoses in children under 13 years old in mainland France was 9.1 per million (CI 95% [5.7 – 12.5]) in 2006. This annual rate was 38 times higher in children born abroad (161.1 per million) than in children born in France (4.2 per million).

## Discussion

Our study provided, for the first time, an estimate of the total number of new HIV diagnoses in children under 13 years old in mainland France during the 2003–2006 period (N = 387). The completeness of the mandatory notification system (DOVIH) and the French Perinatal Cohort (EPF) was under 30%. The observed number of cases in the three linked sources was 56%.

### Limitations and strengths

Possible violations of the underlying capture-recapture assumptions could influence the validity of our outcomes. Our estimates should be interpreted with caution because the criteria of the capture-recapture method have not been fully satisfied [8].

#### Identification of common cases

Record linkage was performed using a combination of identifiers, including the year of birth. A limited number of common cases were identified between registers and were confirmed through subsequent manual validation, thereby minimising the violation of the perfect record-linkage assumption. Links may have been missed between the source LaboVIH and the 2 other sources, potentially resulting in an under- or overestimation of the number of new HIV diagnoses.

#### Closed population

The study period and the geographic area were the same for all of the sources. However, it was estimated that the EPF cohort covered 70% of the HIV-positive pregnant women, which could have introduced a bias, which would result in either an over- or underestimation of our results.

#### Independence between sources

The positive dependence between the DOVIH and EPF sources has been suspected prior to analysis. The heightened awareness of the paediatricians who participate in the EPF cohort of the necessity of reporting to the mandatory notification, as implemented in 2003, may explain this dependence. Two large laboratories participated in both the EPF cohort and the laboratory survey, which could result in a positive dependence between the EPF and LaboVIH sources.

#### Capture homogeneity

Three variables of heterogeneous catchability were identified: country of birth, region of diagnosis and year of diagnosis. The selected model included the 3 variables of heterogeneous catchability and gave an estimate of 387 cases (Table 2), which was slightly higher than the model including dependences between sources only.

### Model selection and estimation

The final model selection in the stratified analysis that included variables of catchability was based on the AIC and DIC, assuming that the goodness of fit of this model, according to the likelihood ratio test, is correct. The approach proposed by Meng and Rubin was applied to utilise the likelihood ratio test and provided p-values slightly lower than the naïve approach (data not shown). AIC/DIC criteria have been obtained by averaging their values over the imputed data sets and therefore should be interpreted with caution [20]. Differences between models according to these criteria may be overestimated and may have led to the selection of an overly complex model.

Model 7 and model 8 give a similar estimate (387 cases). Despite a slightly higher AIC and DIC, we retained model 7 due to its slightly better likelihood statistic (p=0.07). Although model 7 is less parsimonious, it includes a biologically plausible interaction term between EPF and year of diagnosis.

### Estimating missing values

The variable ‘place of birth’ was not recorded in the source LaboVIH but was nearly complete for the two other sources. Typically, the standard approach in a capture-recapture method is to ignore variables not common to every source, which often leads to biased estimates of the population size [24]. One commonly used approach to the analysis of incomplete data sets is to impute missing values and analyse the data set as if it were complete. Such methods of single imputation are not statistically valid, may yield biased estimates, and lead to underestimated variances [25]. Two methods that are currently recommended to handle missing values adequately include the maximum likelihood estimation (MLE) and MI. These methods are asymptotically equivalent and require the same assumption that the data are missing at random (MAR), i.e., the missing data mechanism depends on observed values only [11,26]. In our study, the variable “place of birth” was missing without indication of an underlying mechanism in the LaboVIH source, which implied that the MAR assumption had been met. Only few studies report the imputation of unobserved values in capture-recapture applications. Both MLE, using an Expectation Maximisation (EM) algorithm [24,27,28], and MI were applied in these studies [29]. Van der Heijden *et al.*[28] estimated missing values for variables of heterogeneous catchability that were not collected in all of the sources, such as gender and region of residence. The authors stressed that the Expectation Maximisation (EM) algorithm sometimes involves complex numerical integration, especially during step E (the algorithm computes the expectation of the log-likelihood evaluated using the current estimate for the parameters), and that MI has the advantage of being computationally much simpler for situations with incomplete continuous variables. Zwane *et al.*[29] demonstrated in their study that MI performed well in a capture-recapture application. They estimated missing values for both continuous and categorical variables of heterogeneous catchability and concluded that MI is preferred to MLE in these circumstances. In our study, the incomplete variable was categorical. Although MLE could have been applied, the MI approach was preferred because it could be implemented in most general statistical software.

When building the imputation model, it is recommended to include any variables that may be used in the subsequent analyses [30]. The following variables were complete within our databases and used as predictors: age, sources, year of diagnosis, and region of diagnosis. Because the variable ‘country of birth’ was missing in LaboVIH, the twofold source*covariate interaction terms were not included in the imputation model. Therefore, the imputation process was assumed to be conducted under the assumption of zero correlation between the omitted variables and the outcome. As a result, the estimates associated with these interaction terms could be biased toward zero [30,31].

According to Graham *et al.*[30,32] and White *et al.*[20], it is recommended to generate a number of databases at least equal to the percentage of incomplete cases, or at least 30 databases in our study. Because only one variable was incomplete, we chose to impute a larger number of databases.

One advantage of MI is that the standard errors and CIs of the estimates are directly available as part of the model estimation. A parametric bootstrap approach has been recommended to calculate CIs for the final estimates [33,34]. This method yields asymmetrical CIs and allows one to take model uncertainty into account. Future research should address the possibility of combining this parametric bootstrap approach with MI.

### Estimates of the number of new HIV diagnoses

Among the 89 estimated new HIV diagnoses in children under 13 years old in 2006, 40 occurred in children born in France. This estimate is more than twice the expected annual number of cases cited by Yeni [4]. However, Yeni’s estimate did not take into account women who are not tested for HIV during pregnancy or women who seroconvert during pregnancy following a first negative test. Both scenarios create a much higher risk of transmission from the mother to the unborn baby. Prior to 1994 in France, in the absence of any prevention strategy, the HIV MTCT rate was approximately 20% [35]. Such high-risk situations were identified in a retrospective analysis of children diagnosed with HIV infection at Necker Hospital in Paris [36].

Our capture-recapture findings allowed us to estimate a rate of new HIV diagnoses in children in mainland France in 2006 of 9.1 per million. This rate was 38 times greater for children born abroad than for those born in France. This ratio is higher than that observed in adults; the rate of new HIV diagnoses in adults born abroad is 6.0 per million, compared with 0.6 per million in those born in France [10]. The higher ratio observed in children can be explained by lower access to HIV screening and prevention of MTCT during pregnancy in HIV-endemic countries.

Our results can be compared with data from the United Kingdom because both countries have similarly sized populations (the U.K. has a population of approximately 60 million, including 10 million children), similarly concentrated HIV epidemics and similarly sized foreign-born populations (the U.K.’s foreign-born population is approximately 8% of the total population, with approximately 0.5 million from sub-Saharan Africa). In 2006, the rate of new HIV diagnoses in children under 15 in the U.K. was slightly higher (10.1 per million) than our estimate for France. This discrepancy is likely due to different HIV prevalence rates within the countries of origin of each country’s foreign-born population. In the U.K., the foreign-born population is primarily from Eastern or Southern Africa. The foreign-born population in France is primarily from Western or Central African countries, where the HIV prevalence is lower. As in France, the number of new diagnoses in children in the U.K decreased from 2003 to 2006 (from 148 to 117) and has continued to decline since then [37]. Additionally, as in France, approximately two thirds of children diagnosed as HIV-infected in the U.K. were born abroad [38].

### Completeness

The completeness of the mandatory notification of new HIV diagnoses in children was low (28%) compared with that of the overall DOVIH system for HIV in children and adults (62% in 2004) [10]. This discrepancy could be explained by the compulsory pre-notification from laboratories by microbiologists for adult HIV, which facilitates DOVIH reporting by clinicians. The notification system of HIV infection in children was modified in 2007 to require microbiologists to report new HIV diagnoses in children. However, low completeness and modification of the surveillance system make it difficult to assess potential trends in new HIV diagnoses that have occurred since 2007.

Several hypotheses may explain the low completeness for HIV diagnoses in children in EPF (26%). Approximately 70% of HIV-infected pregnant mothers and their children have been included in the EPF cohort. Cases of HIV-infected children born to mothers who were not included in EPF, and especially to those who delivered abroad, may have been missed for two reasons: (i) data have been collected retrospectively for 2003 and 2004, and (ii) parental consent after HIV diagnosis in children is sometimes difficult to obtain for paediatricians.

## Conclusion

Our study provided, for the first time, an estimated annual rate of new HIV diagnoses in children under 13 years old in mainland France. A more systematic HIV screening of pregnant women that is repeated during pregnancy among women likely to engage in risky behaviour is needed to optimise the prevention of MTCT. The high prevalence of HIV infection in certain regions of the world, especially in sub-Saharan Africa, could justify screening guidelines for children who migrate to France, as is currently recommended for adults. Thus, children diagnosed as HIV-infected would benefit from an early and appropriate treatment. Notification of new HIV diagnoses in children should also be improved to better describe the evolving epidemiology of HIV infection in children.

## Competing interests

The authors declare that they have no competing interests.

## Authors’ contributions

VHB contributed to the study conception, performed statistical analysis and prepared the final draft of the manuscript. AG conceived and supervised the study and helped to draft the manuscript. FL participated in the design and coordination of the study and helped to draft the manuscript. FC, JW, and CL participated to the data gathering and contributed to interpretation of the study. ME participated in the design of the study, contributed to the acquisition of data, and helped to perform the statistical analysis. All of the authors critically revised and approved the final manuscript.

## Pre-publication history

The pre-publication history for this paper can be accessed here:

http://www.biomedcentral.com/1471-2334/12/251/prepub
